# Dr. Darwall on Cerebral and Spinal Irritation

**Published:** 1829-10-01

**Authors:** 


### Periscope;

OR,

### CIRCUMSPECTIVE REVIEW.

"Ore trahit quodcunque potest, atque addit acervo."

I.

### On some Forms of Cerebral and Spinal Irritation. By Dr. Darwall.

A paper on this interesting subject has just appeared in our Midland contemporary, from the pen of Dr. Darwall of Birmingham, which is deserving of notice. There can be no doubt that modern pathologists have contemplated the brain and nervous system as passive rather than active agents in disease, if not in health: that is, they consider that the circulation influences the brain much more than the brain influences the circulation. That there is a reciprocal influence exerted, cannot be questioned, but we have no doubt that infinitely more of our *disorders* and their consequences, *diseases*, arise through the instrumentality of the brain and nervous system than through disturbances of the circulation produced independently of this source. It should never, indeed, be forgotten that the animal functions, as well in health as in disease, operate in a circle—that the interruption may begin in any one part, and be thence communicated to many other parts, which re-act in their turn, on the primary source of disturbance.

After alluding to the discoveries of Bell and Philip, respecting the nervous system, and the influence of the nerves on secretion, Dr. D. thinks we may fairly conclude that, "when indigestion and its accompanying evils of nervous irritability, severely affect any individual, the brain and spinal chord, one or both, are suffering, as well as the stomach."

"But I wish to go further than this point, and to regard the nervous system not as merely influenced by affections of the kind above alluded to, but as actually being the source from which they proceed. It is now much more generally acknowledged than formerly, that functional must precede organic diseases, and consequently, if the nervous system preside over and govern function, this same system must, in very many instances, be first affected. It becomes, then, necessary to ascertain these instances, for it is clear that our chance of curing disease, must be in proportion to the accuracy of our diagnosis in the earliest periods.

"The affections which appear to me referable to irritation of the spinal chord, are, perhaps, in their origin, confined to this portion of the nervous system only; but so intimately are the sympathies united, that but a very short time elapses before the brain is also comprehended in the disorder. Of the nature of the irritation, or what is the state of the blood-vessels of the part affected, dissection cannot afford us any information, inasmuch as patients rarely die from maladies of this kind, till the functional has passed into organic disease. But I am inclined to believe, that in most cases, there is some irregularity in the local circulation—that there is frequently congestion, and it may be conceived, that it will sometimes proceed into acute or chronic inflammation.

"One other circumstance may be referred to before entering upon the consideration of the affections in question; it is simply this, that disorders attacking the origin of nerves, or their attachment to the central mass, whether this be the brain or the spinal chord, always disturb the functions of the organs to which such nerves are destined. It may be imagined that the ramification of nerves may be affected in their passage, and thus, also, give rise to similar phenomena; no reason can be alleged that such a circumstance may not happen, but we know actually that the former does occur, and we may not neglect it in the treatment of diseases, and if by any means we could recognise the latter affections, they also would demand an equal attention. It is of the former, however, which I now propose to treat, and the first class are those in which the functions of the heart suffer.

"That mental anxiety has frequently given rise to organic disease in this viscus, is well ascertained, and the progress of such cases is generally the following. Depression of spirits, with dyspepsia, and occasional palpitation of the heart; considerable debility; great excitability; and every time that this is acted upon, it is followed by exhaustion, and more or less severe hypochondriacism. Emaciation occurs, and generally in proportion to the affection of the heart. After some time, the palpitation becomes more and continual; the action of the heart is perceptible over a larger space, sometimes on both sides of the chest; the pulse at the wrist is various, according to the nature of the cardiac affection; if the ventricles become thin and weak, the pulse is rapid, small, and feeble, undulating; if, on the other hand, active aneurism of the organ exist, the pulse is, in the earlier stages, excessively powerful, and sometimes continues so till within a few hours of death. In other cases, it resembles, for weeks before dissolution, the pulse which attends passive aneurism. Unless death occur suddenly, these affections terminate in general or local dropsy, and the patient dies completely worn out and exhausted.

"In women, the catamenia become very early deranged; they are profuse or suppressed, and in either case attended with severe and profuse leucorrhœa. The following cases appear to me illustrative of these observations. In the first, the disease was removed by early attention: in the second, it is most probable that irremediable organic alterations had taken place."

We shall briefly sketch out these cases.

*Case* 1. *Jan.* 10*th*, 1828. A young man, a jeweller, aged 19 years, applied to our author, having been previously under a respectable surgeon for dyspepsia. He had suffered from moral causes, and was obliged to work fourteen or fifteen hours daily to support his wife and himself. His appetite failed—he became flatulent—had palpitation of the heart, which gradually increased till he could no longer move without exciting violent action of the organ. By attention to the digestive organs the appetite improved, but the palpitation continued. A blister was applied to the upper part of the dorsal vertebræ, which greatly diminished the palpitation. In a week another blister was applied, and soon afterwards the palpitation ceased. Afterwards he appeared to continue well. The only addition to the previous treatment was the application of the blister to the spine.

"The grounds upon which this treatment was instituted, were these. The disease had evidently originated from mental disturbance, acting through the nervous system, and appeared to have affected more particularly that part of it which presides over the functions of the heart. This organ is supplied with nerves partly from the *par vagum*, which takes *its rise from the medulla spinalis*, and partly from the cardiac plexus, of the sympathetic, which receives twigs from the spinal nerves. My object, therefore, was to apply counter-irritation, as near the common centre of cardiac nerves, as lay in my power. Whatever may be thought of the reasoning, the success of the treatment was undoubted: nor, I think, can it be doubted, that it was one of those affections which so frequently terminate in organic disease of the heart."

We are rather surprised to find the par vagum taking rise from the *medulla spinalis*, as we generally have seen it rise from the corpora olivaria of the medulla oblongata. Dr. D. no doubt, considers this part as only the highest point of the medulla spinalis.

The second case is still under our author's care, and is, he fears, irremediable.

*Case* 2. *Dec.* 31*st*, 1828. A young woman, aged 19, has severe and constant palpitation of the heart, with small, quick, fluttering, irregular pulse. The ventricular contractions can be heard in every part of the chest, and the palpitation is increased after taking food, or the slightest exercise. She is flatulent, costive, urine turbid and scanty, catamenia regular but dark coloured —*profuse leucorrhœa* — dyspnœa, but no cough—heaviness and giddiness of head, with noise in the ears—"*pain* in the nape of the neck, increased, and particularly distressing, on the slightest agitation." She describes it more as stiffness and irritation than violent pain —very low spirited. She has been ill for seven years, and her complaints began soon after the impression of triste moral emotions, and the drain of a profuse leucorrhœa. Afterwards the palpitation increased, and it was much aggravated by a fright, after which she was incapable of doing any thing. She still remains with the same state of heart and pulse. The third case we shall give in Dr. D.'s own words.

*Case* 3. "*Nov*. 1828. I visited Mrs. Wilson, and found her labouring under the most violent palpitation of the heart I had ever seen. The bed-clothes were heaved up with each contraction of the ventricle, and the pulse in the carotids was tremendous. On applying the stethoscope to the region of the heart, a very loud hissing noise was distinctly heard, at intervals, corresponding with the pulse at the wrist. The palpitation came on more violently at one time than at another, but had continued at the time of my visit, almost without intermission, for nearly a fortnight. She was very low spirited, and hysterical, but without fever. Her bowels had been purged, and leeches had been applied to the region of the heart, but without benefit. She was again purged, and a blister applied to the spine, which rose in about eight hours, and the palpitation had diminished as soon as the irritation from the blister was perceptible. I saw her the next day, and the palpitation was almost gone. The hissing sound had quite disappeared. It returned slightly a day or two after, but has never again been so violent. Gentle aperients, with the mineral tonics, were given, and in a few days she was able to go to work. She has had many of these attacks within the last few years.

"*December* 6*th*, 1828.—I have seen her this morning, and she is entirely free from inordinate action of the heart. Her pulse was eighty-four and quiet. She refers her relief to the blister."

In the second case, the leucorrhœa, we imagine, tends very much to keep up the inordinate action of the heart. All drains upon the system, especially that of blood from piles, and profuse muco-purulent secretions from the vagina, have a very powerful effect in disturbing the action of the heart, and imitating organic disease there. In the third case, we have also to recollect that the patient was an hysterical female, and we all know what fearful forms hysteria will assume, and how obstinate they will prove at one time, and fugitive at another. Dr. D. observes that there was one symptom in the second case which scarcely leaves a doubt of the medulla spinalis being implicated, viz. "the pain in the nape of the neck." If Dr. Darwall will carefully inquire among his dyspeptic patients, he will find this symptom present in three cases out of four, though it will not always be told to the doctor unless he makes inquiries. Of all sympathetic pains we have found this the most common in dyspepsia, and we really cannot bring ourselves to believe that it depends on an affection of spinal marrow. It is true that, being a sympathetic pain it must be produced through the medium of the brain or spinal marrow; but we do not suppose it is what Dr. D. means by spinal or cerebral irritation.

"Another form in which spinal and cerebral irritation are exhibited, occurs most frequently in young women. It is attended *by severe and constant pain in one or both hypochondria*, extending to the shoulder and arm of the affected side, *not always aggravated by pressure, and ceasing immediately upon or in a short time after lying down*. It is this pain of which the patients generally complain, and it frequently endures for several years. It is generally attended with much constitutional disturbance. *There is head-ache, with great heat on the external surface of the head, severe throbbing of the temples, and pain in the nape of the neck. The temper is capricious, and the spirits very variable*: and at the catamenial periods, the depression, in many cases, is excessive. The tongue, after some time, is furred, and at the back part, greatly tuberculated. The stomach suffers under the various forms of dyspepsia, gastrodynia, flatulence, pyrosis, &c.; the bowels are, in many instances, much distended, and there is continual clangor intestinorum. Palpitation of the heart, more or less, is present, and often dyspnœa attends. The most common disturbance, however, is in the uterine function, and I have scarcely seen an instance in which this has not occurred. Most commonly there is menorrhagia; in some few cases the catamenia are diminished, or they are completely suppressed. But whether they are increased or diminished, leucorrhœa almost invariably attends, and is generally more or less profuse, in proportion to the duration of the general disorder. When the catamenia are profuse, they are usually, in the earlier part of the period, dark coloured and grumous, and are accompanied with severe pain. The urine, for the most part, deposits a light coloured sediment, and varies much in quantity, being occasionally very copious and limpid. The bowels are constipated, and yet no great relief is obtained by purging.

"If this state be suffered to proceed without remedy, emaciation ensues, and great debility, till at length the individual is incapable of following any occupation. Her countenance becomes leucophlegmatic; her ankles swell at night, and without being so ill as to be considered in danger, she settles down into that morbid condition to which the term cachexia has been applied, when every organ suffers in some degree, but none predominates. When this happens, it is generally too late to afford any relief. I have had a young woman under my care for some time, under the last mentioned circumstances. She has continual pains referred to one part or another, but most frequently fixed to a spot under the left breast; she is leucophlegmatic; her ankles are at times œdematous; her breathing short and hurried; her spirits very varying, and she is subject to much head-ache and giddiness. At different times she has received relief from local depletion, and upon the whole, she is better than when I first saw her; but there seems no prospect of affording her any permanent benefit."

Dr. D. mentions the case of a married woman, who had suffered for more than a year, from severe pain in the course of the descending colon. "It was attended with much hysteria." Her medical attendants had purged and leeched their patient, without any good effect—and blisters had been applied to the seat of pain. Dr. D. applied a blister to the lower dorsal and upper lumbar vertebræ. "The next day the pain had nearly disappeared." A second blister completely removed the pain. Her general health was then restored by tonics, gentle aperients, and attention to diet.

"A second class of cases, are those in which the brain appears to suffer considerably, and where, though the catamenia are diminished, or almost suspended, the countenance is pale and leucophlegmatic; the pulse weak; the breath hurried; and the pain in the side varying. Cases of this kind frequently require more active treatment, and in the first instance even admit of general depletion, though this cannot be carried far; indeed, the immediate effect of bleeding in such circumstances, is often an increase of the weakness, and but a very slight relief of the other symptoms. Remedies, nevertheless, appear afterwards to be more efficient, and it has seemed to me, that patients who have suffered depletion, recovered much more quickly than those who were not bled.

"A young woman, two and twenty years of age, had suffered from considerable depression of spirits for several months, when to this, *pain in the side, stiffness in the back of the neck, palpi-*

*tation of the heart, and dyspnœa,* succeeded. There was some leucorrhœa; the catamenia were irregular in their occurrence, and less in quantity; the pulse was feeble; the countenance sallow and anxious. About a fortnight before she applied to me, the left ankle had become painful and œdematous, particularly towards the evening. She had also intense head-ache. She was bled and purged, and for some days remained very feeble; the head was less painful, but was still hot on the surface, and she suffered much from tinnitus aurium; the pain in the side remained the same. Cold applications were made to the head, and a blister placed along the spine, from the nape of the neck to the middle of the back. The next day all her symptoms were relieved; she continued the cold application for a few days, whenever the scalp was warmer than usual, till the tinnitus aurium completely disappeared. After this time, aperients, with the nitro-muriatic acid, were given, and her health improved rapidly. Her appearance now, not more than two months from her being bled, is that of florid health."

The difficulty of breathing, Dr. D. observes, is sometimes so great as, (when the cough is also considered) to induce the fear of organic disease of the lungs. "The stethoscope, in these cases, is a great assistant in the diagnosis. The respiratory murmur is heard in every part of the chest, and is even puerile." This heightening of the respiratory murmur is, Dr. D. thinks, characteristic of the complaint.

"Another variety of this complaint is, where pain in the nape of the neck is complained of, with a sense of pricking and chilliness along the spine. This is most severe at the catamenial period, which, in these cases, is very irregular, and attended with much pain and depression of spirits. Numbness of one or both upper extremities, is sometimes felt, and I have even known complete, though temporary, paralysis occur, which disappeared spontaneously in a few hours.

"To enumerate all the varieties of these affections, would be endless; in all of them there is depression of spirits; in almost all, there is pain and stiffness at the back of the neck, and the nervous sensations are peculiarly distressing. In some, in addition to the symptoms above mentioned, there are slight spasmodic twitches occurring in different parts of the body, and pointing out more decidedly the nervous origin of this disorder."

The last case which our author mentions was that of a female cook, aged 30 years, who had laboured under what was considered to be common dyspepsia both by himself and others. She afterwards became very hysterical—"once or twice had a paralytic loss of power in one arm, and frequently complained of a numbed state of the tongue and sense of fulness in the throat, which disappeared without medical assistance.

"Leeches were applied, but her complaints were aggravated. Her pulse was weak, from 80 to 90; the catamenia scanty; leucorrhœa profuse; her whole appearance leucophlegmatic. To this state frequent attacks of giddiness succeeded, with great depression of spirits, and at length, an attack resembling epilepsy. The common run of alteratives, aperients, &c. were tried in vain. She was taken into the hospital, but came out without any improvement. A this period a seton was placed in her neck, and for some time her health materially improved; her stomach recovered its power, and all her uncomfortable sensations diminished. They have since re-appeared, and her state has been exceedingly variable: there seems now no prospect of a recovery. Since the cerebral symptoms have been more developed, general bleeding has given her temporary relief, but only temporary."

Dr. D. considers the foregoing cases, and cases analogous, as of the same nature as those which Dr. Marshall Hall has denominated, disorders of the general health.

"If, however, they are accurately investigated, it can, I think, be scarcely doubted, that the nervous system suffers in all these examples, primarily and seriously. The intellect is unclouded, but incapable of energetic action; the temper is exceedingly capricious, and the spirits are lowered or elevated far beyond what the circumstances which produce such effects, could lead us to expect; at times, indeed, the mental affections amount almost to insanity; kindness is overlooked or perversely misinterpreted; suspicions are entertained, the most unfounded and the most irrational; the bitterest expressions are employed, without any settled belief, that those deserve them against whom they are directed; and, in short, every thing indicates that state which has most appropriately been called highly nervous. Is it possible that all these things can occur, without affection of the cerebral and spinal system?

"Again, there are local pains, neither exhibiting the symptoms, nor yielding to the remedies of inflammation, but ceasing upon assuming the reclined posture. Knowing, as we do, that the same circumstances occur when pain is experienced in the back from affection of the spine, is it not fair, in defect of other explanations, to refer them also to disturbance of the nervous system? Again, these pains are distant from the spinal chord, but if Sir H. Halford's hypothesis be correct, of one source at least of Neuralgia, viz. irritation of the nerve within the skull, this will serve rather to corroborate the opinion."

Dr. D. has made some judicious observations on the moral treatment of this class of complaints, which deserve attention. The paper indeed contains, as our readers will perceive, many ingenious remarks, and important practical inferences. If counter-irritation to the spine be found as efficacious in dyspepsia, by others as by the author, he will have conferred a great benefit on society.

The next article in the same journal is a case bearing directly on the subject under investigation:—namely, one of severe dyspnœa, succeeded by epilepsy, and cured by counter-irritation of the occiput and back of the neck, by Mr. Romney of Worcester. The patient was a prisoner in the county goal, under sentence of transportation, and was brought from the tread-wheel to the hospital of the prison, on the 23d of March last, with very hurried and feeble respiration, small quick pulse, and great depression of spirits. The countenance was pale, tongue clean, bowels rather confined. No pain, cough, nor mucous rattle on inspiration. The breathing was from 100 to 120 in the minute. Pressure on every part of the abdomen was borne without pain. He was put to bed, and a dose of calomel, with a saline purgative draught given. He passed a sleepless night. The bowels acted freely; but there was no amendment of the symptoms in the morning. Some blood was taken from the arm, and faintness ensued. He passed another sleepless night, from the hurried respiration.

"March 27th.—On repeating the bleeding this morning, although he had no dread of the operation, he suddenly became extremely faint, and respiration being suspended, apparently from spasm of the diaphragm, he had nearly expired. Camphor and opium were now had recourse to in considerable doses, but with no good effect. A dozen leeches were applied to the epigastrium, which bled freely, and he got a warm bath of the temperature of 90 degrees. He then appeared relieved for a few hours, but the hurried breathing returning, the leeches were repeated, followed by the warm bath, and a large blister to the epigastric region; and his bowels which were confined, were relieved by castor oil. In spite of these and similar remedies, the hurried breathing, sleepless nights, and mental despondency, continued with very few and short intermissions till the 26th of April, on which day his respiration suddenly became more hurried than ever, and this distressing affection continued unabated for several days and nights, depriving him almost of the power of speaking, and totally of rest. This state was succeeded by the most violent Epileptic fits I ever witnessed, requiring, with only short intervals, four men to hold him for several days and nights. These fits left him very exhausted and weak, and the respiration remained unimproved. He was in this state when I requested Dr. Malden to visit him, who gave it as his opinion that the peculiar Dyspnœa had, in all probability, depended upon irritation of the cervical portion of the spinal chord, and that the supervention of the epilepsy might be accounted for upon the supposition of this irritation extending to the base of the encephalon. With these views, he recommended the whole back part of his head to be shaved, and covered with a large blister, discharge to be encouraged from it, and before the blister healed, a seton to be put in the back of his neck. These directions were strictly followed. From this time he had no return of epilepsy; the Dyspnœa also gradually went, and he had no relapse, although he remained in the prison some months afterwards."

That the blistering contributed to the amelioration of the symptoms in the above case, we do not deny; but as it is quite evident that there was nothing inflammatory in the complaint from the beginning, we strongly suspect that a "cessation of hostilities"—namely, a hiatus in the depletive measures, was not the least operative part of the methodus medendi. The injurious effects of bleeding and purging in states of great mental depression are not sufficiently known; and we cannot but feel some surprise that, a pale countenance, small pulse, clean tongue, great depression of spirits, freedom from pain on abdominal pressure, and hurried feeble respiration, should not have induced Mr. Romney to pause in the repeated employment of leeches, venesection, and purgation, in such a case. The protraction of the symptoms, and the supervention of epilepsy, shewed abundance of *irritation;* but we believe that there was not a particle of inflammation in the case from beginning to end. God forbid that we should censure Mr. Romney for his practice in this instance. It was not until after we had often committed the same error, that we saw the necessity of caution. A careful and candid review of the case will probably induce Mr. Romney to think that the same course of proceeding would not be advisable under similar circumstances again.

---

